# Customizing 3D Hierarchically Macroporous Covalent Organic Framework Superstructures for Improved Biosensing Interface in Highly‐Sensitive Electrochemical Biosensors

**DOI:** 10.1002/exp2.70141

**Published:** 2026-05-18

**Authors:** Wenbo Wei, Sheng‐Hua Zhou, Dong‐Dong Ma, Qing Li, Mao‐Yin Ran, Shu‐Guo Han, Xiaofang Li, Minghong Huang, Qi‐Long Zhu

**Affiliations:** ^1^ School of Materials Science and Engineering Zhejiang Sci‐Tech University Hangzhou China; ^2^ State Key Laboratory of Structural Chemistry Fujian Institute of Research on the Structure of Matter Chinese Academy of Sciences (CAS) Fuzhou China; ^3^ School of Chemistry and Chemical Engineering Jiangsu University of Technology Changzhou China; ^4^ School of Civil and Environmental Engineering University of Technology Sydney Ultimo New South Wales Australia

**Keywords:** biosensing interface, covalent organic frameworks, electrochemical biosensors, hierarchical nanostructures, ordered macropores

## Abstract

The construction of an optimized biosensing interface is critically important for electrochemical biosensing. Herein, we achieve the controllable construction of macroporous covalent organic framework (COF) nanostructures through a template‐assisted assembly growth strategy, resulting in well‐organized morphologies that are well‐suited as advanced electrode materials. These macroporous structures with unique pore microenvironments support efficient immobilization of biorecognition elements via structure‐confined supramolecular interactions. Notably, the 3D hierarchically macroporous COF superstructure enables the creation of an improved biosensing interface, facilitating enzyme immobilization, enhanced mass and charge transfer, and efficient substrate enrichment, thereby significantly boosting the biosensing performance. Consequently, the developed biosensing electrode loaded with acetylcholinesterase (AChE) displays high sensitivity, stability, and strong anti‐interference capability for the electrochemical detection of diverse organophosphorus pesticides (OPs), with the ultralow limits of detection at a sub‐pg mL^‒1^ level. Moreover, a portable biosensing device integrated with AChE‐loaded HMSCOF demonstrates reliable recovery levels (ranging from 96.4 to 105.3%) for OP detection in river water and leafy vegetables, underscoring its practical applicability. This study demonstrates that the amalgamation of macroporous COF‐based materials with biorecognition elements offers a promising strategy for the advancement of high‐performance portable electrochemical biosensors intended for environmental monitoring and food safety applications.

## Graphical abstract

The 3D hierarchically macroporous COF superstructure, which demonstrates enhanced mass and charge transfer along with superior substrate enrichment at the biosensing interface, serves as an advanced electrode material for enzyme immobilization. The resulting biosensing electrode exhibits excellent sensitivity for the detection of harmful organophosphorus pesticides.

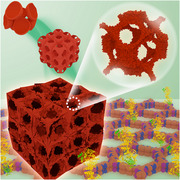

## 1 Introduction

Over the past few decades, electrochemical biosensors have emerged as an extremely competitive and versatile analytical tool for applications in environmental monitoring, food safety testing, medical diagnostics, and biological analysis due to their distinctive advantages, including high selectivity and sensitivity, rapid response, straightforward equipment, and ease of miniaturization [1, 2, 3, 4, 5, 6, 7, 8]. The advancement of electrochemical biosensors relies heavily on the construction of biosensing interfaces between biorecognition components and electronic devices, where the biorecognition elements (e.g., enzymes, nucleic acids, proteins) are assembled onto the electrode materials to transduce biological interactions into readable electronic signals, especially in the case of enzyme‐based electrochemical biosensors [9, 10, 11, 12, 13, 14, 15, 16]. Obviously, the performance of enzyme‐based electrochemical biosensors is largely determined by the careful selection of stable and biocompatible electrode materials with enhanced enzyme loading, which not only facilitates the specific and sensitive biorecognition of target analytes but also ensures the efficient transmission of electrochemical signals. Therefore, the design and synthesis of high‐quality electrode materials are paramount to the development of improved biosensing interfaces in electrochemical biosensors.

Covalent organic frameworks (COFs) have emerged as a highly promising platform in the design and customization of porous organic polymer materials, garnering significant attention in the fields of sensing, gas storage, batteries, and catalysis, owing to their attractive properties, including tunable pore sizes, periodic structures, high specific surface areas, and chemical and thermal stability [17, 18, 19, 20, 21, 22, 23, 24, 25, 26, 27]. In particular, in the construction of stable and reproducible electrochemical biosensors [28, 29, 30, 31, 32, 33], COFs not only facilitate the efficient immobilization of biorecognition elements at the sensing interface of biosensors through self‐supplied non‐covalent interactions (i.e., hydrogen bonding, π‐stacking, hydrophobicity, and electrostatic interactions), but also allow the interface to effectively concentrate analytes, thereby enhancing the detection capability of electrochemical biosensors [34, 35, 36]. However, the conventional synthesis of COFs via uncontrolled covalent self‐assembly of organic building blocks usually results in disordered structures. This leads to aggregated, lump‐like stacking of COFs, which increases mass transport resistance and limits the exposure of active sites due to the long channels and localized mismatched aggregation [28, 37, 38, 39]. These structural issues can compromise the performance of COF‐based electrochemical biosensors, primarily due to the inefficient biosensing interface.

Notably, morphology engineering represents a significant approach for optimizing the applications of COFs [38, 39, 40, 41, 42, 43, 44, 45, 46, 47]. Among these morphologies reported, the thin nanosheets (NSs) are of particular interest due to their ability to fully expose active sites and enhance mass and charge transfer. In a previous work, we developed an interfacial perturbation growth method to obtain the ultrathin (∼1.95 nm) nitrogen‐ and sulfur‐rich bis‐thiazolyl 2D‐COF‐NSs for constructing a high‐performance biosensor [46]. Although the abundant edge‐unsaturated sites of ultrathin 2D‐COF‐NSs facilitate the loading of biorecognition elements, the lack of interlayer mutual support in these structures results in stacking issues during the construction of electrochemical biosensor devices, which in turn diminishes both the mass and charge transfer efficiency at the biosensing interface and the immobilization stability. By contrast, through three‐dimensional (3D) construction to integrate hierarchically porous morphologies and high crystallinity, these COFs encourage the rapid diffusion of guest molecules to the COF surface using the interconnected multilevel pores, providing a feasible solution to this problem [48, 49]. In addition, the macro‐ and mesopores in these 3D structures could serve as nano‐sized reservoirs to enhance the local concentration of analyte molecules [50, 51, 52]. Consequently, we propose that a rational design and assembly strategy for constructing 3D hierarchically porous COF nanostructures would facilitate a more streamlined way to enhance the mass and charge transfer and enriching their edge‐unsaturated sites for enzyme immobilization. Unfortunately, despite the potential advantages, few studies have reported the synthesis of 3D hierarchically porous COFs to date.

In this work, we developed a template‐assisted assembly growth (TAAG) strategy to construct a series of macroporous nanostructures of the bipyridine‐based COF‐TpBpy, which were applied as the advanced electrode materials for electrochemical biosensing. Particularly, the 3D hierarchically macroporous superstructure of COF‐TpBpy (HMSCOF), assembled from ultrathin nanosheets, enables the creation of an improved biosensing interface, facilitating mass and charge transfer and providing abundant free functional groups and edge‐unsaturated sites for effective enzyme immobilization through strong intermolecular interactions. This unique structure makes it an ideal electrode material for boosting its biosensing performance. Consequently, the as‐obtained biosensing electrode assembled with acetylcholinesterase (AChE)‐loaded HMSCOF demonstrates high sensitivity and stability for the selective detection of a wide range of organophosphates (OPs). By contrast, the biosensing electrodes constructed from discrete hollow bowl‐shaped COF (HBCOF) and inter‐stacked macroporous COF (MCOF) counterparts deliver inferior limits of detection. Furthermore, a portable biosensing device was developed using a commercialized screen‐printed electrode (SPE) integrated with AChE‐loaded HMSCOF, demonstrating high sensitivity and reliability for the detection of the actual samples collected from river water and leafy vegetables. Our study highlights the importance of rational biosensing interface design in COF‐based biosensors, underscoring their practical applicability and offering valuable guidance for future advancements in biosensing technology with potential applications in environmental monitoring and food safety.

## 2 Results and Discussion

### 2.1 Material Synthesis and Characterization

**FIGURE 1:**
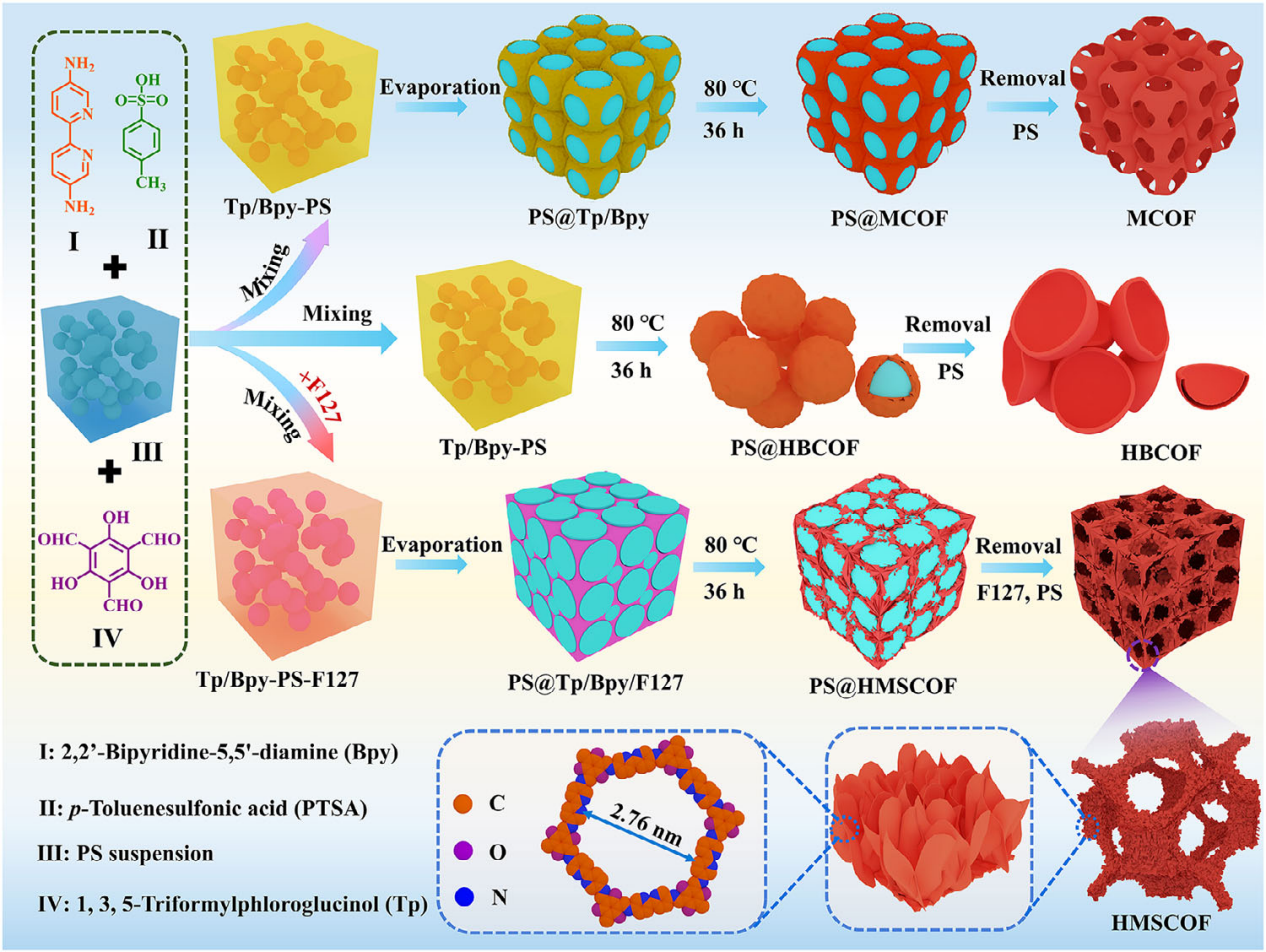
Schematic representation for the preparation of MCOF, HBCOF, and HMSCOF with unique nanostructures.

As shown in Figure 1, the COF nanomaterials with controllable nanostructures were prepared by using the TAAG strategy through the Schiff‐base reaction between 1,3,5‐triformylphloroglucinol (Tp) and 2,2’‐bipyridine‐5,5’‐diamine (Bpy), with the colloidal monodisperse polystyrene (PS) nanospheres as the hard template and *p*‐toluenesulfonic acid (PTSA) as the catalyst. Intriguingly, the modification of the methodology could result in the COF nanomaterials with diverse nanostructures. Especially for achieving the 3D hierarchically macroporous superstructure of HMSCOF, Pluronic poly(ethylene oxide)‐poly(propylene oxide)‐poly(ethylene oxide) (PEO‐PPO‐PEO) triblock copolymer F127 was required as the secondary soft template.

In this typical synthesis of HMSCOF, F127 micelles were firstly assembled on the spherical PS surface (PS‐F127), followed by the sequential addition of the organic linkers and catalyst, which were mixed sufficiently to obtain the Tp/Bpy‐PS‐F127 suspension. Then, the solvent in the suspension was evaporated to form a closely stacked monolith, which was further incubated at 80°C for 36 h to induce the polymerization and crystallization, resulting in the PS@HMSCOF composite. Finally, HMSCOF was successfully obtained by the removal of the PS and F127 templates and unreacted precursors (for more details, see Synthesis in Materials and Methods and Supporting Information). The SEM images of samples at different reaction stages and reaction times clearly demonstrate the growth process of HMSCOF (Supplementary Figures S1 and S2). In this process, the self‐assembly stacking induced by solvent evaporation is important to form the 3D macroporous stacked structure of HMSCOF, while the addition of F127 is an indispensable soft template to generate the hierarchical texture assembled from the ultrathin nanosheets of COF‐TpBpy. For comparison, the similar process to HMSCOF, but in the absence of F127 as the soft template, led to the generation of inter‐stacked macroporous COF‐TpBpy (MCOF). In addition, when the COF‐TpBpy grew rapidly on the PS surface in solution, without the solvent evaporation‐induced self‐assembly process, an independent spherical structure was formed. Subsequent removal of the PS template resulted in the formation of the concave hollow nanospheres due to the lack of support, producing the discrete hollow bowl‐shaped COF‐TpBpy (HBCOF).

**FIGURE 2:**
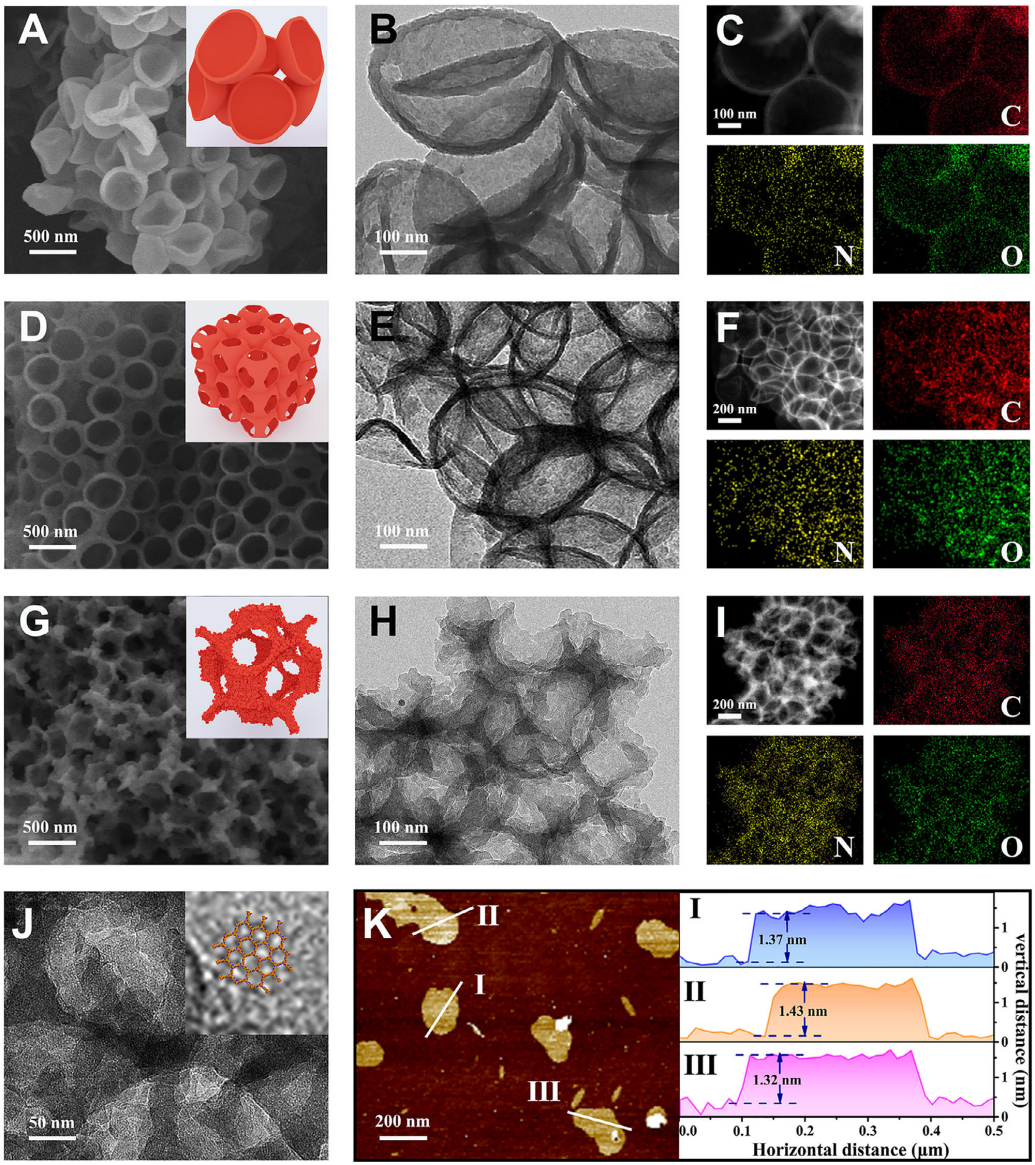
Morphological characterization of the COF nanomaterials: SEM image (inset: structure model), TEM image, HAADF‐STEM images, and corresponding EDX element mapping images for C, N, and O of (a–c) HBCOF, (d–f) MCOF, and (g–i) HMSCOF. (j) HRTEM image (inset: crystalline 2D honeycomb‐like structure of COF‐TpBpy). (k) AFM image and corresponding height profiles of the COF‐TpBpy nanosheets in HMSCOF.

The growth and structure evolution of these COF nanomaterials were studied by scanning electron microscopy (SEM) and transmission electron microscopy (TEM) measurements. As illustrated in the SEM images (Supplementary Figures S1a, b), the PS nanospheres used as the template exhibit uniform particle sizes (∼400 nm) with smooth surfaces. The PS@HBCOF before template etching demonstrates a notable increase in surface roughness as compared to the PS template (Figure S3), which suggests that COF‐TpBpy has been successfully coated on the PS surface. Subsequently, after the removal of the PS template, the resulting HBCOF presents a concave hollow bowl‐shaped nanostructure (Figures 2A, B and Supplementary Figures S7a–c). This phenomenon can be attributed to the absence of interconnectivity between the spherical structures, which renders them incapable of withstanding capillary pressure and consequently leads to their collapse into a concave bowl‐shaped configuration upon the template removal. By contrast, the PS nanospheres in the PS@MCOF composite obtained by solvent evaporation‐induced self‐assembly became a closely stacked monolith with COF‐TpBpy growing within the interstices (Supplementary Figure S4). Then, the removal of the PS template results in the formation of the interconnected and uniformly arranged macroporous cavities in the MCOF (Figures 2D, E, and Supplementary Figures S7d–f). As shown, the macroporous structure of MCOF is composed of the cross‐linked macropores with a diameter of about 300 nm and the surrounding nanowalls with a thickness of about 15 nm. Intriguingly, it is noteworthy that the addition of F127 during the synthesis of HMSCOF resulted in the growth of COF‐TpBpy along the periphery of F127 attached to the PS surface (Supplementary Figure S2), thereby forming the 3D hierarchically macroporous superstructure assembled from the ultrathin nanosheets, as revealed by the SEM and TEM images (Figures 2G, H). In particular, the high‐resolution TEM (HRTEM) image of HMSCOF endorses the ultrathin nanosheets on the macroporous skeleton and well‐defined periodic pore lattice of COF‐TpBpy (Figure 2J). Furthermore, the atomic force microscopy (AFM) image of HMSCOF along with the height profiles demonstrates that the average thickness of the COF‐TpBpy nanosheets is only ∼1.4 nm (Figure 2K). Additionally, the energy‐dispersive X‐ray (EDX) element mapping images show the uniform element distribution throughout the frameworks of these COF nanomaterials (Figures 2C, F, and I). The above results elucidate that the template‐assisted assembly growth of the COFs is an effective strategy for the nanostructure manipulation. Particularly, the unprecedented 3D hierarchically macroporous superstructure of HMSCOF assembled from ultrathin nanosheets could provide significant advantages for enhancing the enzyme immobilization and promoting the mass and charge transfer, which are favorable to achieving the high performance of the resulting electrochemical biosensing electrode. Concurrently, the COF synthesized with only F127 monomicelles as the template for comparison (FCOF) shows aggregated short rods, due to the absence of a PS template for creating macroporous structures (Supplementary Figure S5). On the contrary, the pristine COF‐TpBpy synthesized without a template shows aggregated bulk particles without any characteristic morphology (Supplementary Figure S8).

**FIGURE 3:**
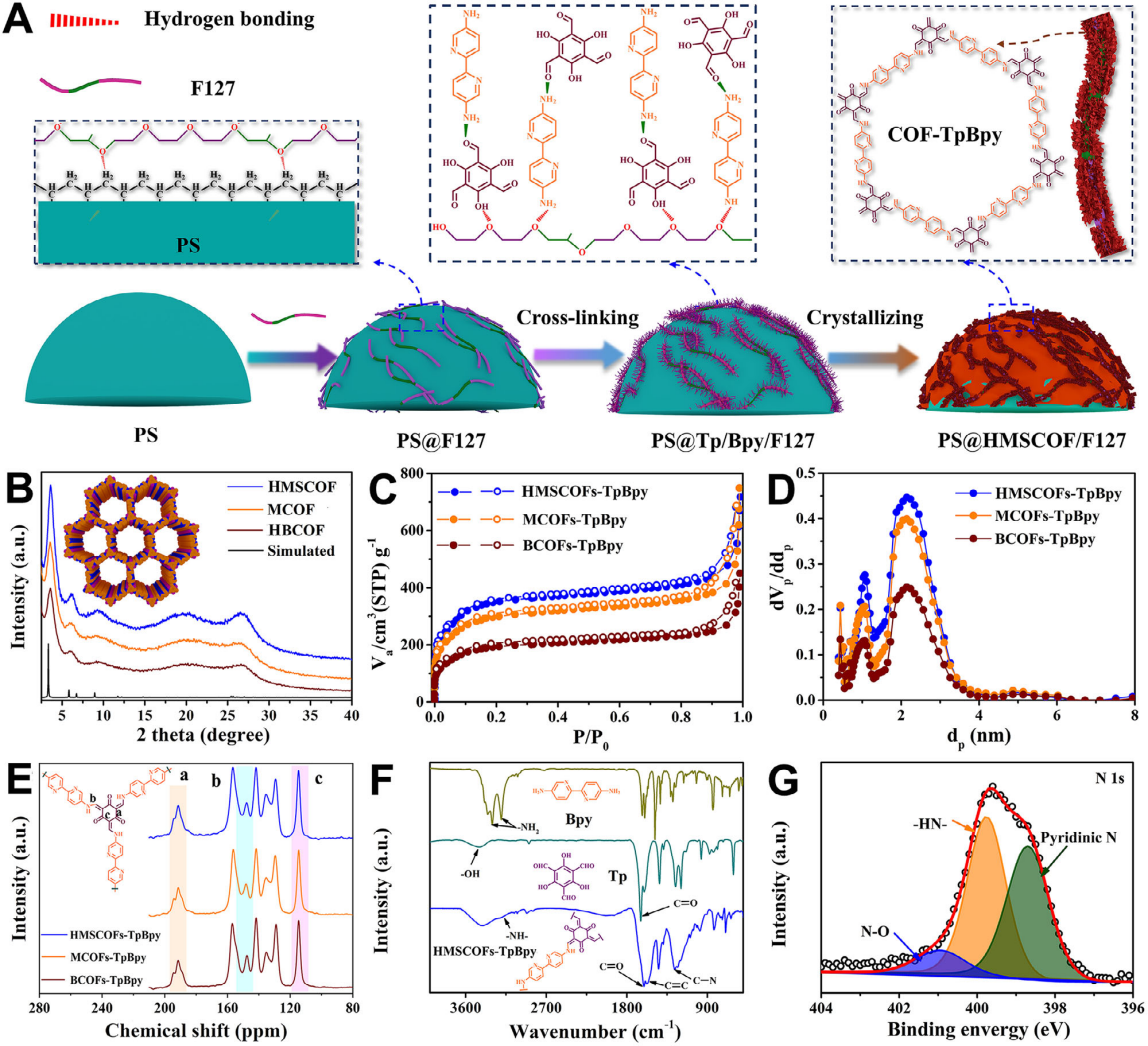
Assembly growth mechanism and structure characterizations of the COF nanomaterials: (a) Schematic illustration of the mechanism for the assembly growth of HMSCOF, (b) Simulated and experimental PXRD patterns (inset: space‐filling packing diagram of COF‐TpBpy), (c) N_2_ sorption isotherms, (d) corresponding pore size distributions, (e) ^13^C CP‐MAS NMR spectra of HMSCOF, MCOF, and HBCOF, (f) FT‐IR spectra, and (g) high‐resolution N 1s XPS spectrum of HMSCOF.

In consideration of the aforementioned observations, we put forth a potential mechanism of the TAAG strategy that facilitates the formation of HMSCOF (Figure 3A). In this synthesis, the oxygen‐containing functional groups on the surface of F127 monomicelles can form abundant weak intermolecular interactions (e.g., hydrogen bonds) with the ‐CH_2_ groups on the PS surface [53, 54], thus obtaining the dual‐template F127/PS composite nanoemulsion (Supplementary Figures S1c, d). During the COF growth, the oxygen‐containing functionalities distributed on the surface of F127 micelles can achieve local enrichment of the organic linkers (i.e., Tp and Bpy) through strong hydrogen bonding, thereby creating a microenvironment favorable for the growth of COF‐TpBpy [55, 56]. Concurrently, this microenvironment can furnish additional sites for the crystallization of COF‐TpBpy and mediate the balance of attractive forces (π‐stacking, van der Waals, etc.) and partition effect for the 2D‐COF layers, thereby providing favorable conditions for the formation of ultrathin nanosheet structures in HMSCOF.

To further recognize crystalline and porous structures of these COF nanomaterials, the corresponding characterization analyses were carried out. As illustrated in Figure 3B, the powder X‐ray diffraction (PXRD) patterns indicate the high crystallinity of HMSCOF, MCOF, and HBCOF, where the intense peak at 3.6° corresponds to the (100) plane of COF‐TpBpy, while the broad peak between 25.1° and 28.6° is assigned to the (001) plane, reflecting the π–π stacking of COF layers [57]. All three COF nanomaterials show the similar PXRD patterns to that of the FCOF and the pristine COF‐TpBpy synthesized without the PS template (Supplementary Figures S6 and S9), manifesting their consistent crystalline structures. A comparison between the experimental PXRD pattern and the simulated one of the eclipsed AA stacking model reveals a high degree of correlation, indicating that the structure of COF‐TpBpy belongs to the hexagonal P6/m space group (*a* = *b* = 29.3 Å, *c* = 3.5 Å, *α* = *β* = 90°, *γ* = 120°) (Supplementary Figure S10 and Table S1) [58]. In addition, the porous structure features of these COFs with different nanostructures were evaluated by the N_2_ sorption at 77 K (Figure 3C). The combined I/IV‐type isotherms, which exhibit high N_2_ adsorption with characteristic hysteresis loops at a higher‐pressure range, evidence the coexistence of micropores and mesopores. The corresponding pore distributions further indicate dual aperture distributions at approximately 1.0 and 2.2 nm (Figure 3D). The apparent pore distribution at 2.2 nm further corroborates the eclipsed AA stacking mode of the COF layers. The Brunauer‐Emmett‐Teller (BET) surface areas of HMSCOF, MCOF, and HBCOF are evaluated to be 1372, 1217, and 741 m^2^ g^−1^, much greater than that (464.9 m^2^ g^−1^) of the pristine COF‐TpBpy (Supplementary Figure S11), indicating that the adjustment of the morphology can effectively improve the porosity. Particularly, the 3D hierarchically macroporous superstructure design of HMSCOF leads to the highest porosity and surface area, potentially improving the pore permeability.

Furthermore, as illustrated in Figure 3E, the solid‐state ^13^C cross‐polarization magic‐angle spinning (CP‐MAS) NMR spectroscopy for all three COF nanomaterials provides the similar signals at 189–194 ppm (carbonyl carbon, providing convincing evidence for enol‐to‐keto tautomerism), ∼147 ppm (C‐NH, amine linkage), and ∼114 ppm (olefin carbon), confirming the successful synthesis of these COFs [59, 60, 61]. The Fourier Transform Infrared (FT‐IR) spectra of HMSCOF show the disappearance of stretching bands based on the N−H bonds (3100‐3400 cm^−1^) of Bpy and the aldehyde group (1641 cm^−1^) of TP. At the same time, the appearance of the stretching vibration peaks of the C = O bond (1611cm^−1^), C = C bond (1576 cm^−1^) and the C−N bond (1267 cm^−1^) provides compelling evidence that the framework structures of these COFs with distinct morphologies were successfully constructed through imine linkages (Figure 3F and Supplementary Figure S12). [60, 61] Meanwhile, the X‐ray photoelectron spectroscopy (XPS) was employed to unveil the chemical states of the elements in HMSCOF (Figures 3G and Supplementary S13). Specifically, the two discernible peaks at the binding energies of 398.33 and 399.82 eV in the N 1s spectrum are associated with the imine N and pyridinic N, respectively, which provides additional evidence for the formation of imine bonds [58, 62, 63]. The thermogravimetric analysis (TGA) of the COFs demonstrates their high thermal stability up to 345°C (Supplementary Figure S14), indicating that the framework structure was successfully established by imines. To determine the general applicability of the proposed TAAG strategy for achieving advanced COF nanomaterials, the COF‐TpBD counterpart was also prepared by substituting Bpy with benzidine (BD), which shows the similar 3D hierarchically macroporous morphology to the bipyridine‐based HMSCOF (Supplementary Figures S15–17).

### 2.2 Electrochemical Biosensing Measurements and Mechanism of Signal Enhancement

**FIGURE 4:**
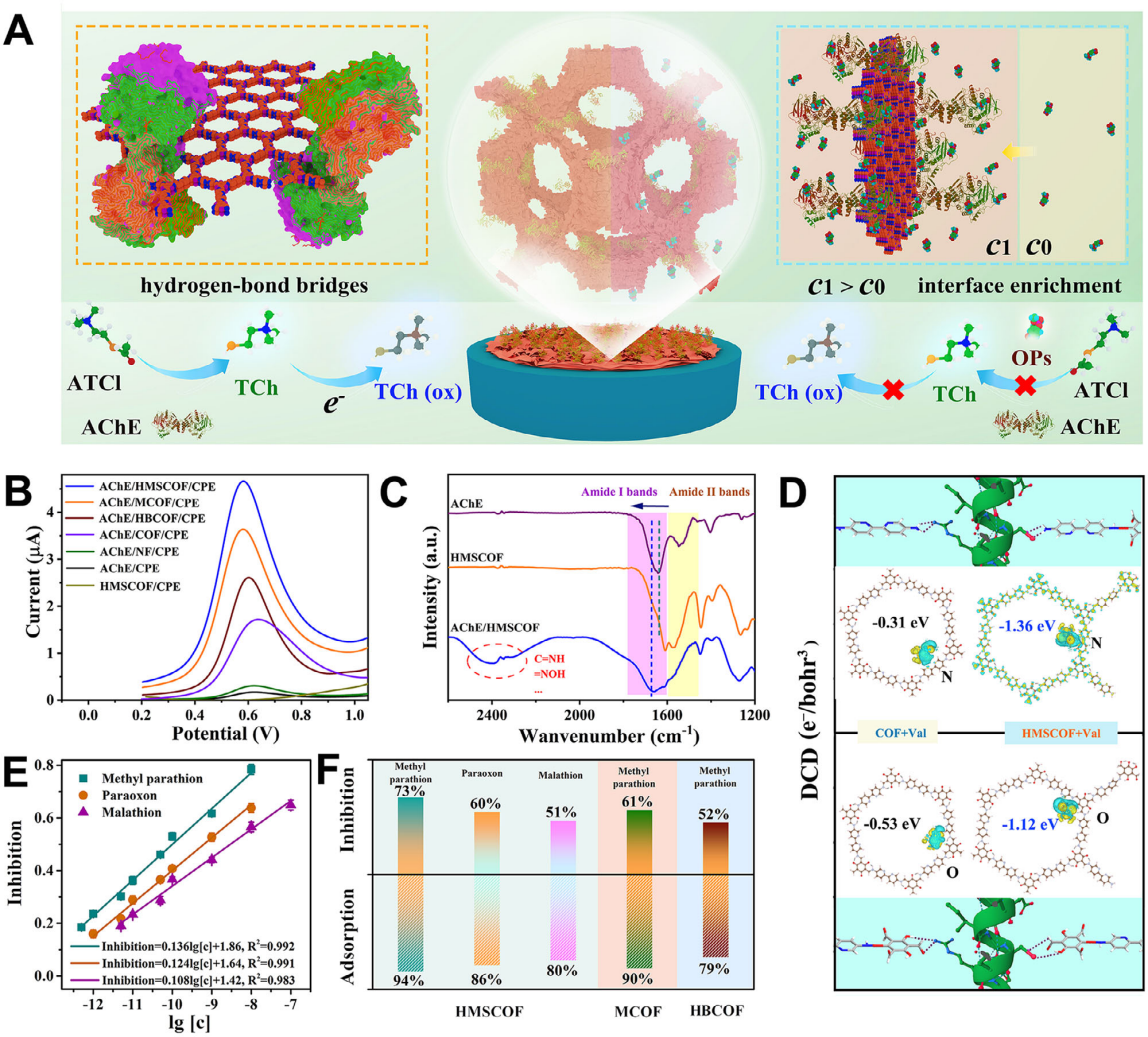
Electrochemical biosensing for OPs: (a) Illustration of the electrochemical biosensing mechanism and the improved biosensing interface facilitating AChE immobilization, mass and charge transfer, and substrate enrichment, (b) DPV curves of different biosensing electrodes in 0.1 M phosphate buffer solution (PBS, pH 7.0) containing 1.5 mM ATCl, (c) FT‐IR spectra of AChE, HMSCOF, and AChE/HMSCOF, (d) Schematic illustration of the hydrogen bonding between the surface residual groups of enzyme protein and the COF organic linkers and the differential charge densities of HMSCOF and COF‐TpBpy after the valine adsorption near the N and O atoms, (e) The inhibition curves of AChE/HMSCOF/CPE for the determination of methyl parathion, paraoxon, and malathion (the error bars are the coefficient of variation across three repetitive experiments), and (f) The inhibition of different modified electrodes toward 50 ng mL^−1^ of methyl parathion, paraoxon, and malathion at 15 min and the adsorption efficiency of different COFs toward 5 µg mL^−1^ of methyl parathion, paraoxon, and malathion at 15 min.

AChE is a highly efficient hydrolase that can catalyze the conversion of acetylthiocholine chloride (ATCl) to thiocholine (TCh) and acetic acid (Figure 4A and Supplementary Equation 1) [64]. TCh is an electroactive molecule that can be oxidized to deliver an irreversible oxidation peak, which can be employed as a marker for pesticide detection (Supplementary Equation 2). As irreversible inhibitors, the OPs can inhibit enzyme activity by forming strong covalent bonds with the active sites of AChE (Supplementary Equation 3). It is evident that the efficient immobilization of AChE onto the electrode materials and the interfacial enrichment of OP analytes are pivotal for enhancing the sensitivity and the detection limit of the biosensing devices. By virtue of the unique nanostructures of the COF nanomaterials, particularly the 3D hierarchically macroporous superstructure of HMSCOF, the biosensing elements prepared with these COFs as the electrode materials provide a favorable biosensing interface for electrochemical biosensing applications. As illustrated in Figure 4A, HMSCOF offers an optimal microenvironment for enhancing AChE loading due to its distinctive superstructure with the ultrathin COF nanosheets on the macroporous skeletons, in which the rich pyridyl, amino, and hydroxyl groups at the nanosheet edges facilitate the formation of robust supramolecular interactions, particularly the hydrogen‐bonding bridges between the surface residues of the enzyme protein and the COF organic linkers. These interactions greatly enhance the immobilization of the enzyme molecules and the maintenance of their biological activity. Furthermore, the porous structures of these COFs with high specific surface areas create advantageous conditions for the interfacial enrichment of the OPs on the surface of the biosensing elements, thus further improving the detection limit. Consequently, the COF‐based biosensing electrodes, prepared by casting the AChE‐loaded COFs onto the carbon paste electrodes (CPE), are anticipated to deliver high electrochemical performance.

The electrochemical performances of these COF‐based biosensing electrodes were evaluated and compared using differential pulse voltammetry (DPV). As illustrated in Figure 4B, all biosensing electrodes prepared with the macroporous COFs (i.e., AChE/HMSCOF/CPE, AChE/MCOF/CPE, and AChE/HBCOF/CPE) exhibit pronounced peak current response at the scan rate of 0.1 V s^−1^ in a 0.1 M phosphate buffer solution (PBS, pH∼7.0) containing 1.5 mM ATCl, which greatly outperforms both pristine COF‐based AChE/COF/CPE and COF‐free AChE/CPE electrodes, highlighting the advantages of the macroporous COF nanostructures. Particularly, the AChE/HMSCOF/CPE electrode delivers the highest peak current in the electrochemical measurement. The superior electrochemical performance of AChE/HMSCOF/CPE can be attributed to the improved biosensing interface endowed by the hierarchically macroporous superstructure of HMSCOF, which significantly improves the enzyme immobilization and the interfacial mass and charge transfer, as well as the enrichment of the analyte molecules. It is noteworthy that the enzyme‐free HMSCOF/CPE exhibits no current response to ATCl, providing substantiation for the biocatalytic role of AChE. Furthermore, in the absence of ATCl, the oxidation peaks are not discernible over AChE/HMSCOF/CPE and other electrodes (Supplementary Figure S18). Meanwhile, the electrode reaction process was investigated by examining the change in current response at varying scan rates in the presence of 1.5 mM ATCl, and the linear plot of current versus potential suggests that the electrode reaction is a surface‐controlled process (Supplementary Figure S19). The electrochemical impedance spectroscopy (EIS) data reveal the progressive influence of the different COFs on the interfacial properties of the electrode (Supplementary Figure S20). The lowest radius in the Nyquist plot and the lowest charge transfer resistance (*R*_ct_) of HMSCOF/CPE indicate the superior charge transfer capacity of HMSCOF. Further, owing to the effective enzyme immobilization, the AChE‐loaded electrode (i.e., AChE/HMSCOF/CPE) well maintained the excellent charge transfer capacity, which is crucial for enhancing the electrochemical response of the resulting biosensing electrode. Moreover, in order to gain a more profound comprehension of the immobilization of AChE on the surface of the HMSCOF‐based electrode, we conducted the investigation on the interactions between AChE and HMSCOF using the FT‐IR spectroscopy (Figure 4C). It is well established that the amide I (1700‐1600 cm^−1^) and amide II (1600‐1500 cm^−1^) bands are distinctive vibration modes of the peptide backbone, which are frequently employed to assess the chemical information pertaining to the peptide backbone [65]. As illustrated, the amide I and amide II peaks are clearly observed in the FT‐IR spectra of both AChE and AChE/HMSCOF, ascertaining the successful immobilization of AChE. As compared to AChE, the peak of amide I for AChE/HMSCOF appears markedly redshifted, indicating the existence of strong hydrogen bonding between AChE and HMSCOF (Figure 4C). The density functional theory (DFT) calculations were employed to further explore the interaction mechanism between AChE and HMSCOF. As shown in Figure 4D, the Bader charge analysis further revealed the much lower adsorption energy of the active sites in HMSCOF with the protein residues (amino acids, represented by valine), which thereby promotes the immobilization of AChE.

As the components of the biosensing electrodes and the testing conditions are of great consequence in determining the quality of the analytical performance, the optimal parameters for electrochemical biosensing experiments were established (Supplementary Figure S21). Then, the reliability for consistent and specific electrochemical biosensing applications was assessed. The AChE/HMSCOF/CPE electrodes prepared in six batches display similar currents with a relative standard deviation (RSD) of only 3.9% in 0.1 M PBS (pH∼7.0) containing 1.5 mM ATCl (Supplementary Figure S22a), indicating good reproducibility of the electrode preparation process. Meanwhile, the initial peak current responses of this electrode were maintained at 97.8%, 95.3%, 96.6%, 94.3%, and 92.1% after storage for 1, 2, 3, 4, and 5 weeks, respectively (Supplementary Figure S22b), demonstrating its long‐term stability. Moreover, the interference tests confirmed the high specificity and anti‐interference capability of the AChE/HMSCOF/CPE electrode, likely due to the ability of the loaded AChE to selectively catalyze the hydrolysis of ATCl even in the presence of 0.5 mM glucose, H_2_C_2_O_4_, citric acid, ascorbic acid, SO_4_^2−^, NO_3_^−^, Na^+^, and Cu^2+^ (Supplementary Figure S23).

Given the notable electrochemical behavior of AChE/HMSCOF/CPE, its biosensing performance for the detection of a range of OPs (e.g., methyl parathion, paraoxon, and malathion) was evaluated under optimal conditions. The biosensing of OPs relies on the inhibition of AChE activity, where the OP molecules bind to the enzyme's active site, preventing it from hydrolyzing ATCl. As the concentration of OPs increases, the enzyme inhibition becomes more pronounced, leading to a reduction in the generation of electroactive TCh. This inhibition is reflected in a decrease in the corresponding peak current in electrochemical measurements, as the lower enzyme activity results in reduced electrochemical signal generation. Therefore, the decrease in peak current serves as an indirect indicator of OP concentration. It was found that there are good linear relationships between the inhibition and lg[c_OPs_] of methyl parathion, paraoxon, and malathion over the AChE/HMSCOF/CPE electrode (Figure 4E). Remarkably, the linear detection ranges for methyl parathion, paraoxon, and malathion were determined to be 5.0 × 10^‒13^ ‒ 1.0 × 10^‒8^, 1.0 × 10^‒12^ ‒ 1.0 × 10^‒8^ and 5.0 × 10^‒12^ ‒ 1 × 10^‒7^ g mL^‒1^, respectively, corresponding to the ultralow detection limits of 0.12, 0.48, and 2.87 pg mL^‒1^ for each OP. In order to gain further insight into the impact of the interfacial enrichment effect on the biosensing performance, we conducted a comparative analysis of the adsorption efficiency and inhibition rate of HMSCOF for methyl parathion, paraoxon, and malathion. It can be observed that HMSCOF exhibits notable adsorption efficiency for these OPs, with a particularly high value of 94% for methyl parathion, resulting in the highest corresponding inhibition rate (Figure 4F and Supplementary Figure S24). This result verifies that the interfacial enrichment effect of the biosensing electrode effectively concentrates the local OPs, thereby improving the detection sensitivity. Furthermore, the comparative results of the adsorption efficiency and inhibition rate of methyl parathion by different COFs, along with theoretical calculations, corroborate this assertion (Figure 4F and Supplementary Figure S25).

### 2.3 Real Sample Detection

**FIGURE 5:**
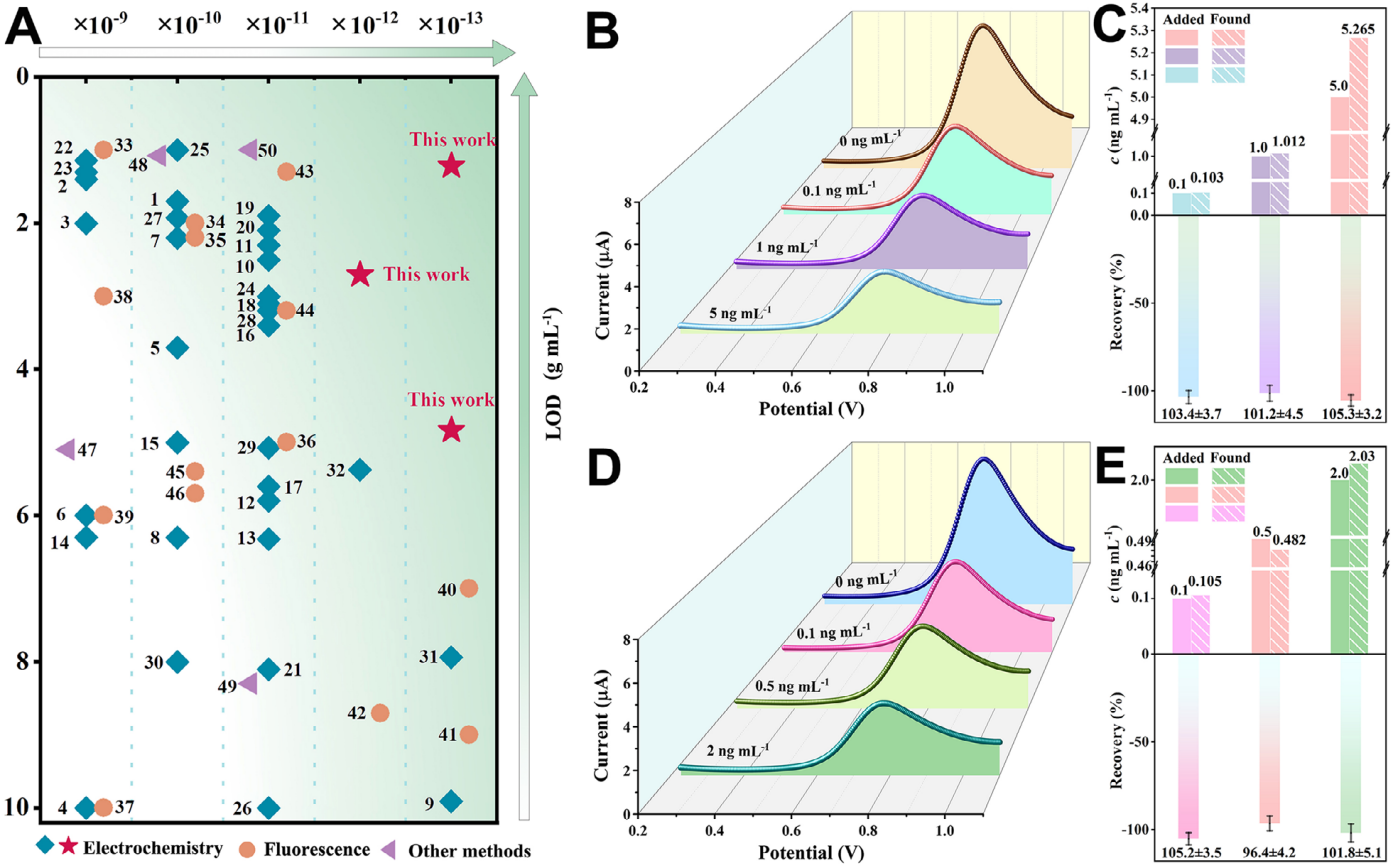
Comparative evaluation and practical application of the AChE/HMSCOF‐based electrochemical sensor: (a) Comparison of analytical performances of AChE/HMSCOF/CPE with other recently reported sensing systems, (b–e) Real sample analysis of AChE/HMSCOF/SPE for (b, c) leafy vegetables, and (d, e) river water with different concentrations of methyl parathion.

Noteworthy, when compared with previously reported sensing systems (Figure 5A and Supplementary Table S2), the electrochemical biosensing performance of AChE/HMSCOF/CPE exhibits broader detection ranges and lower detection limits for a series of OPs. Consequently, HMSCOF proves to be an ideal electrode material for biosensing due to its superior structural characteristics and electrochemical performance, thus providing a solid foundation for the development of portable and field‐deployable biosensing devices. To evaluate the practical capability, a portable biosensing device constructed with the AChE/HMSCOF/SPE electrode was applied for detecting authentic water samples through a comparative analysis of the OPs concentration in river water and leafy vegetables (Supplementary Figure S26a). Initially, the electrochemical biosensor was calibrated using artificial water samples within the concentration range of 1.0 × 10^‒11^ to 1.0 × 10^‒8^ g mL^‒1^ (Supplementary Figure S26b). Subsequently, the practical experiments were carried out by spiking methyl parathion into river water and leafy vegetable extracts at varying concentrations, followed by the electrochemical biosensing measurements (Figures 5B, D), which yielded satisfactory recovery rates ranging from 96.4 to 105.3%, as reported in Figures 5C, E. Moreover, the testing data from this biosensing device were in excellent agreement with those measured by high‐performance liquid chromatography‐tandem mass spectrometry (HPLC‐MS) (Supplementary Tables S3, S4). The results highlight the potential of our biosensing device for the on‐site detection of methyl parathion contamination in water and food, demonstrating its feasibility for field applications in OPs detection.

## 3 Conclusions

In summary, a series of macroporous COFs with diverse pore architectures were successfully developed by using a TAAG strategy for electrochemical biosensing applications. Remarkably, the 3D hierarchically macroporous COF superstructure, comprising innumerable ultrathin COF nanosheets on the macroporous skeletons, provides a favorable biosensing interface that promotes the formation of robust supramolecular interactions. This greatly enhances the immobilization of enzyme molecules while preserving their biological activity. Moreover, the hierarchically porous structure with a high specific surface area significantly improves the mass and charge transfer and creates favorable conditions for the analyte enrichment at the interface, thus enhancing the detection limit. As a result, the biosensing electrode, assembled with AChE‐loaded HMSCOF, displays an outstanding performance for the electrochemical detection of OPs (e.g., methyl parathion, paraoxon, and malathion), achieving unprecedented sensitivity, stability, and ultralow detection limits at the sub‐pg mL^‒1^ level. The mechanism underlying the signal enhancement for electrochemical biosensing of OPs was also elucidated. Additionally, the portable biosensing device integrated with AChE‐loaded HMSCOF exhibits high sensitivity and reliability for the analysis of real‐world water samples. This study could pave the way for the future development of hierarchically porous COF materials in electrochemical biosensing, with promising applications in environmental and food monitoring and beyond.

## 4 Experimental Section

### 4.1 Synthesis of HMSCOF

In a clean 15 mL vial, 5 mL of colloidal PS with a solid content of ∼10‐12 wt% and 100 µL of F127 colloid were added and mixed thoroughly. Furthermore, p‐toluenesulfonic acid (PTSA, 500 mg, 2.5 mmol) and 2,2'‐bipyridine‐5,5'‐diamine (Bpy) powder (84 mg, 0.45 mmol) were added and mixed well in a vortex shaker for 10–15 min. Subsequently, 63 mg of 1,3,5‐triformylphloroglucinol (Tp, 0.3 mmol) was added, and the mixture was vortexed for several minutes until a distinct color change was observed, from cream to orange‐yellow. Subsequently, the mixture was poured into an open Petri dish and left to stand at room temperature for 24 h. Following the evaporation of the water, the mixture was transferred to an oven at 80°C and held for 36 h. Subsequently, the samples (PS@HMSCOF) were thoroughly washed with hot water in order to remove the PTSA and then subjected to a Soxhlet extraction with tetrahydrofuran (THF) in order to remove the PS and F127 template, thus obtaining HMSCOF. The corresponding macroporous COFs, designated MCOF, were synthesized using only PS colloids as templating agents. Furthermore, the synthesis of the corresponding macroporous COFs, designated HBCOF, was conducted using PS colloid as the sole template agent and without standing at room temperature.

### 4.2 Electrochemical Measurements

The AChE/HMSCOF/CPE was employed for the determination of pesticides using the differential pulse voltammetry (DPV) method. The performance of the biosensor was tested by its DPV response in pH 7.0 PBS solution containing 1.5 mM ATCl. Then the electrode was rinsed with water and incubated in an aqueous solution containing the desired concentration of pesticides (methyl parathion, paraoxon, and malathion) for 15 min. Finally, it was transferred into the 1.5 mM ATCl solution for DPV measurements under the same condition.

### 4.3 Measurement Procedure

The estimation formula of the limit of detection (LOD) was calculated as follows:

(1) $$\mathrm{LOD}=K\times S_{b}/m$$

where *S*_b_ is the standard deviation (SD) of the response of the blank solution (without the analyte), m is the slope of the analyte in the linear detection range, and K is a numerical factor selected based on the expected confidence level (*K* = 3 is used, allowing a confidence level of 99.86%).

The inhibition rate of pesticides was calculated as follows:

(2) $$\mathrm{Inhibition}\left(\%\right)=\left(i_{p,\mathrm{control}}-i_{p,\exp}\right)/i_{p,\mathrm{control}}\times 100\%$$

where *i*_p,control_ is the peak current of ATCl over AChE/HMSCOF/CPE, *i*_p,exp_ is the amperometric response of ATCl on AChE/HMSCOF/CPE with pesticide inhibition. Inhibition (%) was plotted against the concentrations of the pesticides to obtain linear calibration graphs.

## Funding

This work is supported by the National Key Research and Development Program of China (2021YFA1500402); the, National Natural Science Foundation of China (NSFC) (52332007, 22575218 and 22175174); and The Science Foundation of Zhejiang Sci‐Tech University (24212185‐Y and 24212183‐Y).

## Conflicts of Interest

The authors declare no conflicts of interest.

## Supporting information




**Supporting File 1**: exp270141‐sup‐0001‐SuppMat.docx.

## Data Availability

All data of this work are present in the article and Supporting Information. The other data that support the findings of this work are available from the corresponding author upon reasonable request.
