# Supplementary material for: Customizing 3D Hierarchically Macroporous Covalent Organic Framework Superstructures for Improved Biosensing Interface in Highly‐Sensitive Electrochemical Biosensors
Source: Exploration (Beijing). 2026 May 18;6(4):70141. doi: 10.1002/exp2.70141 (PMC13394959; doi:10.1002/exp2.70141)
Supplement: Supplementary file 1 [file EXP2-6-70141-s001.docx]

Supplementary Information for

**Customizing 3D hierarchically macroporous covalent organic framework superstructures for improved biosensing interface in highly-sensitive electrochemical biosensors**

**1. Experimental section**

**1.1 Materials**

All chemicals were purchased from commercial sources and used without further treatment. Acetylcholinesterase (AChE, from Electrophorus electricus, lyophilized powder, 200-1,000 units mg^−1^, 2 KU), acetylthiocholine chloride (ATCl), Nafion solution (5 wt%) and Pluronic® F127 were obtained from Sigma-Aldrich. Hexamethylenetetramine, phloroglucinol, trifluoroacetic acid, hydrochloric acid, benzidine, 5-Amino-2-chloropyridine, 2,5-hexanedione, triethylamine, tetraethylammonium iodide, dibromobis (triphenylphosphine) nickel (II), zinc powder, 1,4-dibromobenzene, 4-aminophenylboronic acid pinacol ester, tetrakis (triphenylphosphine) palladium, 1,2-dibromoethane, were purchased from Aladdin Industrial Corporation. Methyl Alcohol (99.9%, Water≤50 ppm), dimethyl sulfoxide-D6 (D, 99.8%), chloroform-D (D, 99.8%), p-Toluenesulfonic acid monohydrate and hydroxylamine hydrochloride were obtained from Adamas. NaOH, K_2_S_2_O_8_, KH_2_PO_4_, K_2_HPO_4_, acetone, [methanol](javascript:;), ethanol, thiourea, tetrahydrofuran, and chloroform were obtained from Sinopharm Chemical Reagent Co. Ltd. (Shanghai, China). Polyvinyl pyrrolidone (PVP, MW 55000) and Styrene (99%) was obtained from Aladdin Co., and the Styrene further was washed by NaOH (5 wt%) aqueous solution to remove the stabilizing agent (p-tert-butylcatechol). K_2_S_2_O_8_ was recrystallized before use. Deionized water was generated by the Hitech purified water system (18.2 MΩ cm).

**1.2 Characterization**

**Transmission electron microscopy (TEM) and high-resolution TEM (HRTEM):** the TEM and HRTEM characterizations were carried out on TALOS F200X G2 field emission transmission electron microscope (ThermoFisher Scientific) at an acceleration voltage of 200 kV. Aberration-corrected high angle annular dark-field scanning transmission electron microscope (AC-HAADF-STEM) was performed with a Titan Cubed Themis G2 300 (FEI) high-resolution transmission electron microscope operated at 200 kV.

**Scanning electron microscopy (SEM):** the SEM characterization was performed on a Carl Zeiss Sigma 300 instrument (operated at an accelerating voltage of 3 kV).

**Nuclear Magnetic Resonance Spectroscopy (NMR):** the ^1^H NMR spectra were recorded on an ECZ400S 400 MHz spectrometer. Solid-state ^13^C CP/MAS NMR spectra were recorded on 400WB AVANCE III (Bruker-BioSpin) plus 400 MHz spectrophotometer at 298 K.

**Powder X-ray diffraction (PXRD):** the PXRD was performed on a Rigaku Miniflex II X-ray powder diffractometer with Cu Kα radiation (λ = 1.54 Å). The 2θ scan range was 2 to 40° at a scan rate of 3° min^–1^.

**Brunauer-Emmett-Teller (BET):** after dehydrated under vacuum at 100 °C for 12 h, the BET specific surface areas of the samples were performed with N_2_ adsorption-desorption isotherms at liquid nitrogen temperature using automatic volumetric adsorption equipment (Belsorp-max).

**X-ray photoelectron spectroscopy (XPS):** the XPS experiments were performed in a Thermo Fischer ESCALAB 250Xi X-ray photoelectron spectrometer with monochromatic Al K_α_ radiation (*hv* = 1486.2 eV), and the binding energies were calibrated by C 1s to 284.8 eV.

**Fourier-transform infrared (FT-IR) spectroscopy:** the FT-IR spectra were recorded with KBr pellets using VERTEX70 infrared spectrometer from Bruker.

**1.3 Synthesis**

**Synthesis of 1,3,5-triformylphloroglucinol (Tp)** ^[1]^

**Scheme S1.** Synthesis of 1,3,5-triformylphloroglucinol.

Phloroglucinol (6.0 g, 49 mmol), hexamethylenetetramine (15.1 g, 108 mmol) and trifluoroacetic acid (90 mL) were mixed in a clean 15 mL flask and refluxed at 100 °C under N_2_. Following a 2.5-hour period, 3 M HCl (150 mL) was added to the reaction solution at a slow rate, and the mixture was heated at 100 °C for an additional hour. After cooling to room temperature, the solution was filtered through Celite, extracted with an excess of CH_2_Cl_2_, and dried over MgSO_4_. The solution was then evaporated under reduced pressure to obtain the product as a light-yellow solid. Yield: 1.52 g (14.8%). ^1^H NMR (400 MHz, d_6_-DMSO, 298 K, TMS): 9.99 (s, 3H), 6.88 (s, 3H).^[1]^

**Synthesis of 5,5’-Diamino-2,2’-bipyridine (Bpy)**^[1]^

**Scheme S2.** Synthesis of 5,5’-Diamino-2,2’-bipyridine.

**1-(2-Chloropyridine)-5-yl-2,5-dimethyl-1H-pyrrole (1)**: in a 250 mL two-necked flask were placed p-TsOH (100 mg, 0.53 mmol), 5-Amino-2-chloropyridine (5.0 g, 39 mmol), 2,5-hexanedione (6 mL, 51.3 mmol) and 80 mL toluene. Then, the resulting solution was heated up to 100 °C for 5 h in a Dean-Stark apparatus. After cooling to room temperature, the reaction mixture was extracted with ethyl acetate several times and washed with NaHCO_3_ solution and brine, dried over Na_2_SO_4_, filtered, and the solvents were evaporated to afford the crude product. Then it was purified by column chromatography (hexane/EtOAc/Et_3_N = 10/1/0.05) on silica gel to give the product as yellow solid 1 (6.7 g, 89%). 1H NMR (d_6_-DMSO, 400 MHz): δ (ppm) 8.37 (d, 1H), 7.86-7.83 (m, 1H), 7.63-7.66 (d, 1H), 5.82 (s, 2H), 1.95 (s, 6H).

**5,5’-Bis(2,5-dimethyl-1H-pyrrole)-2,2’-bipyridine (2)**: tetraethylammonium iodide (6.4 g, 25 mmol), NiBr_2_(PPh_3_)_2_ (3.7 g, 5 mmol) , zinc powder (2.78 g, 42.5 mmol) and 10 mL absolute THF were added in a 250 mL three-necked flask. The flask was evacuated and refilled with argon for three times. Then, the mixture was heated to 60 °C with stirring for 3 h under the protection of N_2_. After that, 30 mL of a THF solution with 1 (5.20 g, 25 mmol) was added dropwise into the mixture and then keep stirring overnight. After cooling down to room temperature, concentrated ammonia (25 wt%, 100 mL), water (50 mL), and DCM (200 mL) were added. The mixture was stirred for another 15 min, then it was filtered and the aqueous phase was extracted with DCM. The extracted organic layers were washed with brine, and the solvents were evaporated under reduced pressure. The crude product was purified by column chromatography (hexane/EtOAc/Et_3_N = 10/1/0.05), giving the light yellow solid. Yield: 3.2 g (77%). 1H NMR (CDCl_3_, 400 MHz): δ (ppm) 8.59-8.56 (m, 2H), 7.73-7.70 (m, 1H), 5.97 (s, 2H), 2.09 (s, 6H).

**5,5’-Diamino-2,2’-bipyridine (3)**: a mixture of 2 (2.38 g, 6.96 mmol), hydroxylamine hydrochloride (14.6 g, 210 mmol), water (20 mL), ethanol (50 mL) and triethylamine (8.0 mL) was heated under reflux for 20 h. After that, the hydroxylamine hydrochloride (14.6 g, 210 mmol) and triethylamine (4 mL) were added into the above reaction solution. Then, the reaction system was refluxed for another 24 h and quenched by 30 mL ice-cold 3 M aqueous hydrochloric acid. The suspension of yellow precipitate was mixed with 100 mL ethanol and fully precipitated in the fridge overnight. The yellow precipitate was filtered out and dissolved in water, subsequently basified with 2 M sodium hydroxide aqueous solution. The solution was extracted with excess CHCl_3_, then the organic layers were dried over K_2_CO_3_, filtered and evaporated to give the light yellow final product 3 (0.57 g, 44%). 1H NMR (d_6_-DMSO, 400 MHz): δ (ppm) 7.89-7.82 (m, 4H), 6.94-6.92 (m, 2H), 5.31 (s, 4H).

**Synthesis of PS template** ^[2]^

Monodisperse polystyrene spheres were synthesized using a typical emulsion polymerization. Typically, ultrapure water (100 mL, 18 MΩ cm^–2^) and styrene (11.8 mL) were added into a three-necked round-bottom flask (250 mL). The mixture was stirred at 600 rpm while heating to 75 °C and purged with nitrogen gas at a flow rate of 60 mL min^−1^. After the mixture was kept at 75 °C for 30 min, K_2_S_2_O_8_ (0.04 g) was added and the reaction was continued for 24 h. The obtained suspension were centrifuged at 4000 rpm to remove any large agglomerates at the bottom and the upper suspension were dried at 60 °C overnight to obtain an ordered PS monolith.

**Synthesis of F127**^[3]^

The aqueoussolution of triblock copolymer Pluronic F127 (1.47 wt %, 60 mL) was stirred at 67-70 °C for about 30 min to obtain short rods of F127 monomicelles.

**The preparation of the carbon paste electrode (CPE)**^[4]^

0.6 g of liquid paraffin and 3.4 g of graphite powder were hand-mixed to produce a homogenous paste. Then the prepared carbon paste was firmly packed into a PVC tube (3 mm internal diameter) and a copper wire (1.5 mm external diameter) was introduced into the other end for electrical contact.

**The preparation of AChE/HMSCOF/CPE modified electrode**

Firstly, 2 mg of HMSCOF powders and 200 μL of 0.5 % nafion (NF) solution were dispersed into 800 μL deionized water under sonication for 30 min to obtain homogeneous suspension of HMSCOF. Then 6 μL of above suspension was cast onto the surface of a freshly polished CPE to obtain HMSCOF/CPE, which was dried at room temperature to form a stable film. Furthermore, AChE/HMSCOF/CPE was prepared by casting 6 μL of AChE solution (0.2 mg mL^–1^) onto HMSCOF/CPE and coated with 5 μL of 0.5 % NF as the protective membrane. Finally, the AChE/HMSCOF/CPE was stored in 0.1 M pH 7.0 PBS at 4 °C in a refrigerator.

**1.4 Electrochemical measurements**

Electrochemical experiments were performed with a CHI660D electrochemical workstation (Shanghai Chenhua) in a conventional three-electrode cell. The saturated calomel electrode (SCE) was served as the reference electrode, a platinum wire electrode was used as the auxiliary electrode, and the different modified electrodes were used as the working electrodes.

Cyclic voltammetric measurements were performed in an undivided 30 mL electrochemical teflon cell at room temperature. AC impedance experiments were carried out in 5.0 mM K_3_Fe(CN)_6_/K_4_Fe(CN)_6_ (1:1) containing 0.1 M KCl, while the applied perturbation amplitude was 0.005 V, the frequencies swept from 10^5^ to 10^–2^ Hz and the initial potential was 0.20 V. The electrolyte was deoxygenated with highly-purity nitrogen for 30 min and maintained under nitrogen atmosphere during measurements.

**1.5 Measurement procedure**

a. Inhibition mechanism of the AChE/HMSCOF electrode by OPs:

**1.6 Computational details**

In our work, DFT calculations were performed in the Vienna ab initio simulation package (VASP).^[5]^ The projector augmented wave (PAW) method was adopted to describe interactions between ions and electrons. The generalized gradient approximation (GGA) in the form of Perdew, Burke, Ernzerhof (PBE) was used to describe electron exchange and correlation.^[6]^ The plane-wave basis set along with a kinetic cutoff energy was 400 eV. The Brillouin zones were sampled with 3×3×1 Monkhorst-Pack meshes. The structures were fully relaxed until the maximum force on each atom was less than -0.02 eV/Å and 10^–5^ eV.

**1.7 Safety precautions and waste disposal**

Appropriate personal protective equipment including lab coats, safety goggles, and gloves was used throughout all experiments. All procedures involving (mention specific hazardous materials, e.g., organic solvents, strong acids/bases, toxic compounds) were conducted in a well-ventilated fume hood. The generated waste, such as (mention specific waste, e.g., solvent waste, toxic compounds), was collected separately and disposed of according to our institution's established environmental health and safety protocols.

**2. Supplementary figures and tables**

**Figure S1.** SEM images of PS spheres, PS-F127, Tp/Bpy-PS-F127, PS@Tp/Bpy/F127.

**Figure S2.** SEM images of PS@Tp/Bpy/F127-0h, PS@Tp/Bpy/F127-6h, PS@Tp/Bpy/F127-12h, PS@Tp/Bpy/F127-24h, PS@Tp/Bpy/F127-48h.

**Figure S3.** SEM image of PS@HBCOF.

**Figure S4** SEM images of PS@MCOF.

**Figure S5** SEM images of Tp/Bpy-F127, FCOF@F127, FCOF.

**Figure S6.** PXRD patterns of FCOF.

**Figure S7.** SEM images and structure model of HBCOF and MCOF.

**Figure S8.** SEM images of COF-TpBpy.

**Figure S9.** PXRD patterns of COF-TpBpy.

**Figure S10.** (a) unit cell and (b) eclipsed crystal lattice packing of COF-TpBpy.

**Table S1.** Fractional atomic coordinates for the unit cell of COF-TpBpy.

| Hexagonal, P6/m  a= b= 29.9 Å, c= 3.5 Å  α = 90.0°, β = 90.0°, γ = 120° | | | |
| --- | --- | --- | --- |
| C1 | 0.05717 | 0.03206 | 0 |
| C2 | 0.02511 | 0.05684 | 0 |
| C3 | 0.10858 | 0.06268 | 0 |
| N4 | 0.14381 | 0.0437 | 0 |
| C5 | 0.1984 | 0.07685 | 0 |
| C6 | 0.23039 | 0.05547 | 0 |
| C7 | 0.28375 | 0.08746 | 0 |
| C8 | 0.30489 | 0.14105 | 0 |
| N9 | 0.27288 | 0.16105 | 0 |
| C10 | 0.22078 | 0.13053 | 0 |
| C11 | 0.36113 | 0.17599 | 0 |
| N12 | 0.38051 | 0.2279 | 0 |
| C13 | 0.43203 | 0.26178 | 0 |
| C14 | 0.46695 | 0.24387 | 0 |
| C15 | 0.44805 | 0.19071 | 0 |
| C16 | 0.39508 | 0.15661 | 0 |
| N17 | 0.52094 | 0.27997 | 0 |
| C18 | 0.55902 | 0.26462 | 0 |
| C19 | 0.60991 | 0.29806 | 0 |
| C20 | 0.63173 | 0.35484 | 0 |
| O21 | 1.04617 | 1.10379 | 0 |
| O22 | 0.76975 | 0.39682 | 0 |

**Figure S11.** (a) N_2_ sorption isotherms and (b) the corresponding pore size distribution of COF-TpBpy.

**Figure S12.** FT-IR spectra of Bpy, Tp, COF-TpBpy, HBCOF, and MCOF.

**Figure S13.** XPS spectra of HMSCOF (O 1s a and C 1s b).

**Figure S14.** TG spectra of HMSCOF, MCOF, HBCOF, and COF-TpBpy.

**Figure S15.** Schematic representation for the preparation and structure and SEM image of COF-TpBD.

**Figure S16.** PXRD patterns of COF-TpBD.

**Figure S17.** (a) N_2_ sorption isotherms and (b) the corresponding pore size distribution of COF-TpBD.

**Figure S18.** DPVs of different modified electrodes without ATCI in 0.1 M pH 7.0 PBS.

**Figure S19.** CV curves of AChE/HMSCOF/CPE in 0.1 m PBS containing 1.5 mm ATCl at scan rates from 0.02 to 0.3 V s^–1^ (Insets: linear dependence of *i*_p,a_ on scan rates).

**Figure S20.** EIS measurements for the different modified electrodes. The plot shows the overlay of the experimental data and the simulated Nyquist plots fitted using the equivalent circuit. The simulated Nyquist plot fitted using an equivalent circuit representing the determination of the solution resistance (R_s_), and resistance to the charge transfer (R_ct_) with schematic of the equivalent circuit used for fitting the Nyquist plots obtained from EIS measurements.

**Figure S21.** Effect of (a) HMSCOF dosages, (b) AChE amounts and (c) pH values on response currents. (d) inhibition toward 5 ng mL^–1^ of methyl parathion at different incubation time by DPV in 0.1 M PBS containing 1.5 mM ATCl.

**Figure S22.** The reproducibility of the biosensor: (a) histograms of amperometric responses of the six electrodes, and (b) stability of AChE/HMSCOF/CPE after 5 weeks.

**Figure S23.** The interference tests of AChE/HMSCOF/CPE.

**Figure S24.** Effect of time on adsorption efficiency of methyl parathion.

**Figure S25.** The differential charge densities of COF after the methyl parathion, paraoxon and malathion were adsorbed near the N.

**Figure S26.** (a) Schematic diagram of the portable biosensor for OPs. (b) DPV curves of AChE/HMSCOF/SPE for the detection of methyl parathion (insets: inhibition curve)

**Table S2.** Comparisons of the reported biosensors for detection OPs.

**Table S2** **(continued).** Comparisons of the reported biosensors for detection OPs.

**Table S2 (continued).** Comparisons of the reported biosensors for detection OPs.

**Table S2 (continued).** Comparisons of the reported biosensors for detection OPs.

**Table S3.** Detection of real samples by this biosensor

**Table S4.** Detection of real samples by HPLC-MS

***References***

[1] Q. Sun, B. Aguila, J. Perman, N. Nguyen, S. Ma, *J. Am. Chem. Soc.* **2016**, *138*, 15790-15796.
[2] K. Shen, L. Zhang, X. Chen, L. Liu, D. Zhang, Y. Han, J. Chen, J. Long, R. Luque, Y. Li, B. Chen, *Science* **2018**, *359*, 206-210.
[3] X. Xi, D. Wu, L. Han, Y. Yu, Y. Su, W. Tang, R. Liu, *ACS nano* **2018**, *12*, 5436-5444.
[4] S. Dong, N. Li, G. Suo, T. Huang, *Anal. Chem.* **2013**, *85*, 11739-11746.
[5] G. Kresse, J. Furthmüller, *Phys. Rev. B* **1996**, *54*, 11169.
[6] J. P. Perdew, K. Burke, M. Ernzerhof, *Phys. Rev. Lett.* **1996**, *77*, 3865.
[7] X. Luo, Z. Luo, X. Wei, L. Jiao, Q. Fang, H. Wang, J. Wang, W. Gu, L. Hu, C. Zhu, *Anal. Chem.* **2022**, *94*, 1390-1396.
[8] K. Niu, Y. Zhang, J. Chen, X. Lu, *ACS Sens.* **2022**, *7*, 3551-3559.
[9] F. Arduini, S. Cinti, V. Caratelli, L. Amendola, G. Palleschi, D. Moscone, *Biosens. Bioelectron.* **2019**, *126*, 346-354.
[10] W. Ruankham, T. Tantimongcolwat, K. Phopin, J. Bausells, M. Hangouët, M. Martin, N. Zine, A. Errachid, *Sens. Actuat. B Chem.* **2022,** *372*, 132614.
[11] K. Niu, P. Sun, J. Chen, X. Lu, *Anal. Chem.* **2022**, *94*, 17177-17185.
[12] S. Nagabooshanam, S. Roy, A. Mathur, I. Mukherjee, S. Krishnamurthy, L. M. Bharadwaj, *Sci. Rep*. **2019**, *9*, 19862.
[13] I. Seymour, T. Narayan, N. Creedon, K. Kennedy, A. Murphy, R. Sayers, E. Kennedy, I. O’Connell, J. F. Rohan, A. O’Riordan, *Sensors* **2021**, *21*, 3149.
[14] J. Wang, W. Lv, J. Wu, H. Li, F. Li, *Anal. Chem.* **2019,** *91*, 13831-13837.
[15] L. Zhou, X. Zhang, L. Ma, J. Gao, Y. Jiang, *Biochem. Eng. J.* **2017**, *128*, 243-249.
[16] J. Wu, Q. Yang, Q. Li, H. Li, F. Li, *Anal. Chem.* **2021**, *93*, 4084-4091.
[17] T. Itsoponpan, C. Thanachayanont, P. Hasin, *Sens. Actuat. B Chem.* **2021,** *337*, 129775.
[18] L. Ren, W. Feng, F. Hong, Z. Wang, H. Huang, Y. Chen, *Food Chem.* **2022,** *386*, 132712.
[19] S. H. Wen, H. Zhang, S. Yu, J. Ma, J. J. Zhu, Y. Zhou, *Anal. Chem.* **2023**, *95*, 14914-14924.
[20] Z. Sun, W. Wang, H. Wen, C. Gan, H. Lei, Y. Liu, *Anal. Chim. Acta.* **2015**, *899*, 91-99.
[21] L. Hou, X. Zhang, M. Kong, G. Jiang, Y. Sun, W. Mo, T. Lin, F. Ye, S. Zhao, *Microchim. Acta.* **2020**, *187*, 204.
[22] G. Zhao, B. Zhou, X. Wang, J. Shen, B. Zhao, *Food Chem.* **2021**, *354*, 129511.
[23] S. Bilal, M. Mudassir Hassan, M. Fayyaz Ur Rehman, M. Nasir, A. Jamil Sami, A. Hayat, *Food Chem.* **2021**, *346*, 128894.
[24] A. Cioffi, M. Mancini, V. Gioia, S. Cinti, *Environ. Sci. Technol.* **2021**, *55*, 8859-8865.
[25] Y. Zhu, Y. Cao, X. Sun, X. Wang, *Sensors* **2013**, *13*, 5286-5301.
[26] L. Liu, D. Xu, Y. Hu, S. Liu, H. Wei, J. Zheng, G. Wang, X. Hu, C. Wang, *Food Control.* **2015**, *53*, 72-80.
[27] S. Du, X. Wang, X. Sun, Q. Li, *Anal. Lett.* **2012**, *45*, 1230-1241.
[28] X. Sun, Y. Zhu, X. Wang, *Sensors* **2011**, *11*, 11679-11691.
[29] X. Sun, Q. Li, X. Wang, S. Du, *Anal. Lett.* **2012**, *45*, 1604-1616.
[30] J. Hu, P. Wen, Y. Wang, J. Yang, Z. Xiao, Z. Xu, Y. Shen, H. Wang, B. D. Hammock, *Food Control.* **2024**, *162*, 110423.
[31] Z. H. Wang, A. S. Viana, G. Jin, L. M. Abrantes, *Bioelectrochemistry* **2006**, *69*, 180-186.
[32] W. Wei, S. Zhou, D. D. Ma, Q. Li, M. Ran, X. Li, X. T. Wu, Q. L. Zhu, *Adv. Funct. Mater.* **2023**, *33*, 2302917.
[33] H. Li, Q. Lu, J. Shi, X. Zhang, P. Sun, X. Yan, G. Lu, *Adv. Funct. Mater.* **2024**, *34*, 2309383.
[34] X. Yan, T. Wang, H. Li, L. Zhang, H. Xin, G. Lu, *ACS Nano* **2022,** *16*, 18421-18429.
[35] C. Sun, N. Liu, J. Liu, T. Lv, C. Yang, C. Su, N. Zhang, H. Li, X. Yan, *Sens. Actuat. B Chem.* **2023,** *375*, 132924.
[36] P. Wang, H. Li, M. M. Hassan, Z. Guo, Z. Z. Zhang, Q. Chen, *J. Agr. Food Chem.* **2019,** *67*, 4071-4079.
[37] J. Mehta, S. Dhaka, A. K. Paul, S. Dayananda, A. Deep, *Environ. Res.* **2019**, *174*, 46-53.
[38] B. Lin, Y. Yan, M. Guo, Y. Cao, Y. Yu, T. Zhang, Y. Huang, D. Wu, *Food Chem.* **2018**, *245*, 1176 -1182.
[39] C. Zhang, P. Du, Z. Jiang, M. Jin, G. Chen, X. Cao, X. Cui, Y. Zhang, R. Li, A. M. Abd El-Aty, J. Wang, *Anal. Chim. Acta* **2018**, *999*, 123-131.
[40] C. Zhang, Z. Jiang, M. Jin, P. Du, G. Chen, X. Cui, Y. Zhang, G. Qin, F. Yan, A. M. Abd El-Aty, A. Hacimüftüoğlu, J. Wang, *Food Chem.* **2020**, *326*, 126813.
[41] F. Zhao, Y. Yao, X. Li, L. Lan, C. Jiang, J. Ping, *Anal. Chem.* **2018**, *90*, 11658-11664.
[42] P. Zhang, T. Sun, S. Rong, D. Zeng, H. Yu, Z. Zhang, D. Chang, H. Pan, *Bioelectrochemistry*, **2019**, *127*, 163-170
[43] R. H. Wang, C. L. Zhu, L. L. Wang, L. Z. Xu, W. L. Wang, C. Yang, Y. Zhang, *Talanta*, **2019**, *205*, 120094.
[44] N. Li, R. Li, Q. Wang, Y. Yang, X. Sun, G. Wang, Z. Li, *J. Hazard. Mater.* **2021,** *415*, 125752.
[45] Y. Liu, T. Li, G. Yang, Y. Deng, X. Mou, N. He, *Chin. Chem. Lett.* **2022,** *33*, 1913-1916.
[46] Y. Shen, X. Gao, H. Chen, Y. Wei, H. Yang, Y. Gu, *J. Hazard. Mater.* **2023,** *451*, 131171.
[47] X. Ge, P. Zhou, Q. Zhang, Z. Xia, S. Chen, P. Gao, Z. Zhang, L. Gu, S. Guo, *Angew. Chem. Int. Ed.* **2020,** *59*, 232-236.
